# DPred_3S: identifying dihydrouridine (D) modification on three species epitranscriptome based on multiple sequence-derived features

**DOI:** 10.3389/fgene.2023.1334132

**Published:** 2023-12-15

**Authors:** Jinjin Ren, Xiaozhen Chen, Zhengqian Zhang, Haoran Shi, Shuxiang Wu

**Affiliations:** ^1^ Key Laboratory of Ministry of Education for Gastrointestinal Cancer, School of Basic Medical Sciences, Fujian Medical University, Fuzhou, Fujian, China; ^2^ Fujian Key Laboratory of Tumor Microbiology, Department of Medical Microbiology, Fujian Medical University, Fuzhou, Fujian, China; ^3^ Institute of Applied Microbiology, Research Center for BioSystems, Land Use, and Nutrition (IFZ), Justus-Liebig-University Giessen, Giessen, Germany

**Keywords:** dihydrouridine, machine learning, *Escherichia coli*, *Schizosaccharomyces pombe*, *Saccharomyces cerevisiae*

## Abstract

**Introduction:** Dihydrouridine (D) is a conserved modification of tRNA among all three life domains. D modification enhances the flexibility of a single nucleotide base in the spatial structure and is disease- and evolution-associated. Recent studies have also suggested the presence of dihydrouridine on mRNA.

**Methods:** To identify D in epitranscriptome, we provided a prediction framework named “DPred_3S” based on the machine learning approach for three species D epitranscriptome, which used epitranscriptome sequencing data as training data for the first time.

**Results:** The optimal features were evaluated by the F-score and integration of different features; our model achieved area under the receiver operating characteristic curve (AUROC) scores 0.955, 0.946, and 0.905 for *Saccharomyces cerevisiae*, *Escherichia coli*, and *Schizosaccharomyces pombe*, respectively. The performances of different machine learning algorithms were also compared in this study.

**Discussion:** The high performances of our model suggest the D sites can be distinguished based on their surrounding sequence, but the lower performance of cross-species prediction may be limited by technique preferences.

## Introduction

The first RNA modification was reported in 1951, and currently, at least 170 types of RNA modifications have been identified among all life domains (Boccaletto et al., 2022). Among these modifications, dihydrouridine (D) is the second most popular tRNA modification (Machnicka et al., 2014), which was introduced as the natural component of yeast tRNA in 1965 (Holley et al., 1965). Additionally, D is conserved in the D-loop of tRNA in Bacteria, Eukaryota, and some Archaea based on mass spectrometry (Kowalak et al., 1995). In recent studies, it has been observed that D has several molecular functions and participates in many biological processes, such as the spatial configuration of RNA, evaluation (Song et al., 2023), cancer development (Xing et al., 2004; Kasprzak et al., 2012), and virus replication. Additionally, the potential associations between SNP and D in disease development were revealed (Song et al., 2023).

The hydrogenation of the uridine C5–C6 bond is regulated by dihydrouridine synthase (DUS) enzymes, which are from a conserved gene family COG0042 (Kasprzak et al., 2012). Each family member is responsible for dihydrouridylation of one or two U positions in a tRNA molecule (Xing et al., 2004). Interestingly, the mRNA expression is associated with the DUS expression based on the knockdown experiment (Kato et al., 2005). The cross-linking and immunoprecipitation (CLIP) analyses also showed that DUS can bind with mRNA (Mitchell et al., 2013). These results suggest that D not only appears in tRNA but also in mRNA.

With the advance in sequencing techniques, the concept of epitranscriptome arose in 2011 (Jia et al., 2011). Multiple methods have been developed in the past 10 years to help decipher the epitranscriptome landscape of different modifications (Dominissini et al., 2016; Yang et al., 2017; Koh et al., 2019). Rho-seq (Finet et al., 2022) is the first D epitranscriptome profiling method based on the reverse transcription arrest. The results of Rho-seq reported hundreds of D sites and suggested the mRNA D modification affects meiotic chromosome segregation. In another study, D-seq (Draycott et al., 2022) was also developed with a similar concept of Rho-seq. In addition to the NGS platform, nanopore techniques could be used to detect RNA modifications, including D sites (Wang et al., 2023a; Song et al., 2023; Zhang et al., 2023).

**FIGURE 1:**
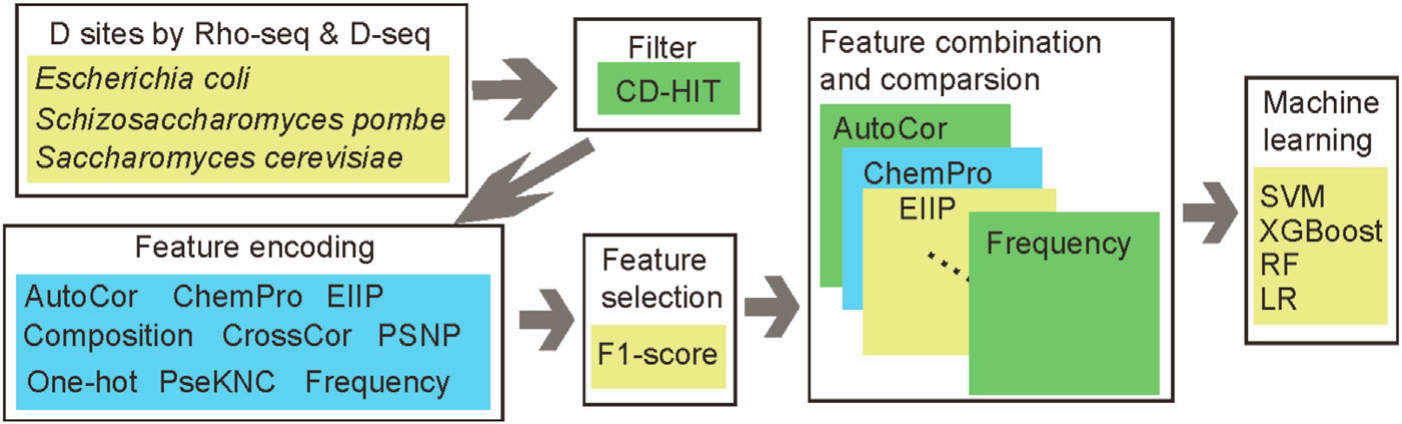
Workflow for DPred_3S. The information on D sites was obtained by Rho-seq or D-seq and filtered by CD-HIT to reduce sequence redundancy. Different feature encoding methods were integrated with their importance and combined together to find the optimal features for D prediction. The different machine learning algorithms were compared in this work also.

Although the sequencing method can provide a precise and accurate location of D modification, the experiment is still time-consuming and expensive. The bioinformatics prediction provides another convenient method to detect putative modification sites. There are some studies providing prediction tools for D identification (Xu et al., 2019; Dou et al., 2021); however, there are two limitations in those studies. First, the number of D sites is limited; only 176 sites were identified by the LC/MS method among five species. Second, these works only considered the D modification of tRNA. To address these, we provided a new prediction framework “DPred_3S” to support the prediction of D sites in three species epitranscriptome. After features and parameter optimization, our models achieved credible performances. The workflow for DPred_3S is summarized in Figure 1. The project code and training sequences are available at https://github.com/SXWuFJMU/Dpred_3S/.

## Methods and materials

### Putative D sites from Rho-seq and D-seq

The processing data were obtained from the original paper. There are 106 and 372 D sites identified in the epitranscriptome of *Escherichia coli* and *Schizosaccharomyces pombe*, respectively (see Table 1). To select positive samples, the sequence length 41 bp of D was primarily used to extract sequence information, which is widely used in many previous studies (Chen et al., 2019a; Liu and Chen, 2020; Song et al., 2020; Liu et al., 2021; Xu et al., 2021). The unmodified uridines were randomly selected from the transcriptome and extended 20 bp in both directions as negative samples. The ratio of positive and negative samples is 1:1. To remove redundant sequences, CD-HIT (Fu et al., 2012) software with default parameters was used to keep the sequence similarity less than 85%. For model training and cross-validation, 80% samples were used and the remaining 20% were considered independent testing data.

**TABLE 1:** Identified D sites by Rho-seq or D-seq.

	D sites	CD-HIT	Training	Test
*Escherichia_coli*	106	57	45	12
*Schizosaccharomyces pombe*	372	247	198	49
*Saccharomyces cerevisiae*	178	176	140	36

### Feature encoding and selection

Sequence-derived features were widely used in the bioinformatics prediction, such as RNA-binding proteins, RNA modification, microRNA interaction, and RNA sub-location. Some recent works have summarized the commonly used encoding features in the bioinformatics prediction field (Hou et al., 2019; Liu, 2019; Su et al., 2020; Chen et al., 2021a). In this study, we considered eight types of encoding methods in the beginning to find the optimal features of D site prediction.

### Binary encoding method

Binary encoding is known as one-hot encoding (ONE_HOT). Each nucleic acid was converted into a four numeric vector based on the following settings: A = (1,0,0,0), U = (0,1,0,0), G = (0,0,1,0), and C = (0,0,0,1).

#### Chemical property

In the chemical property (ChemProper), the ring structure, functional groups, and hydrogen bonds of nucleic acids were considered to be the features. A and C have the amino group, whereas G and U have the keto group. In hybridization, A and U have two hydrogen bonds, but G and C have three hydrogen bonds, and A and G have two ring structures, whereas C and U only have one. Based on these concepts, each nucleic acid can be presented as three numeric vectors as

$$\left\{\begin{array}{l} \begin{array}{lll} A & = & \left(1,1,1\right) \end{array}\\ \begin{array}{l} \begin{array}{ccc} U & = & \left(0,0,1\right) \end{array}\\ \begin{array}{lll} G & = & \left(0,1,0\right) \end{array}\\ \begin{array}{lll} C & = & \left(1,0,0\right). \end{array} \end{array} \end{array}\right.$$

#### Electron–ion interaction pseudopotentials

The electron–ion interaction pseudopotentials (EIIPs) were proposed by Veljko and Dragutin (Lalović and Veljković, 1990), and each nucleic acid can be represented by a number due to their electron–ion interaction pseudopotentials. The A, U, G, and C values equal to 0.1260, 0.1335, 0.0806, and 0.1340, respectively.

#### Nucleic acid composition (CONPOSI)

The frequency of each dinucleotide is calculated, which can be presented as a vector with 16 numbers:

$$f=\left(f_{AA,}\;f_{AU,}\;f_{AC,}\ldots \ldots .\;f_{UG,}f_{UU}\right).$$

#### Accumulated nucleotide frequency (frequency)

This encoding method considered the position and order of nucleic acids. In a sequence, the frequency of nucleotide in the i -th position is equal to the sum of all the instances of the i -th nucleotide before the i+1 position divided by position i , which can be summarized as the following formula $f_{i}=d_{i}/i$ .

#### Auto-correlation (autoCor) and cross-correlation (crossCor)

These two methods were invented based on the physicochemical (PC) properties between two nucleotides. autoCor considers the correlation coefficient of the same PC properties between two subsequences, whereas crossCor focuses on the correlation coefficient of the different PC properties between two subsequences. More detail information was introduced in previous studies (Song et al., 2022).

#### Pseudo k-tuple composition (PseKNC)

PseKNC is the most popular encoding method which was used in multiple types of bioinformatics prediction, including but not limited to protein, DNA, and RNA prediction (Chen et al., 2013; Lin et al., 2014; Chen et al., 2018). The PseKNC section in the webserver iLearnPlus (Chen et al., 2021b) was used in this project to generate sequence-derived features.

In feature optimization, the F-score (Chen and Lin, 2006) was used to evaluate the discriminative capability in the i -th position. $\left(+\right)$ and $\left(-\right)$ presented the features were from positive samples and negative samples, respectively.

$$F_{i}=\frac{{\left({\bar{x}}_{i}^{\left(+\right)}-{\bar{x}}_{i}\right)}^{2}+{\left({\bar{x}}_{i}^{\left(-\right)}-{\bar{x}}_{i}\right)}^{2}}{\frac{1}{n^{+}-1}\sum_{d=1}^{n^{+}}{\left({\bar{x}}_{d,i}^{\left(+\right)}-{\bar{x}}_{i}^{\left(+\right)}\right)}^{2}+\frac{1}{n^{-}-1}\sum_{d=1}^{n^{-}}{\left({\bar{x}}_{d,i}^{\left(-\right)}-{\bar{x}}_{i}^{\left(-\right)}\right)}^{2}}$$

In addition, based on the order of F-score, the incremental feature selection (IFS) (Lin et al., 2014) was used to identify the optimal features.

### Machine learning algorithms and evaluation

Support vector machine (SVM) is a widely used machine learning approach in bioinformatics research. In this study, SVM with default parameters from LIBSVM (R language interface) was used in feature optimization (Chang and Lin, 2011). To evaluate the impact of machine learning algorithms, generalized linear model (GLM), random forest (RF), and naive Bayes (NB) from the R package caret were used to compare the performances from different methods (Kuhn, 2008). Finally, we analyzed the regularization parameter C and the kernel width parameter γ in SVM to select the optimal parameter for our model.

$$\left\{\begin{array}{l} 2^{-5}\leq C\leq 2^{15}\;with\;step\;of\;2\\ 2^{-15}\leq y\leq 2^{5}\;with\;step\;of\;2^{-1} \end{array}\right.$$

To evaluate the performances, AUROC (area under the receiver operating characteristic curve) was used as the key evaluator. AUPRC (area under the precision-recall curve) was calculated in SVM parameter optimization. The accuracy (ACC), sensitivity (Sn), and specificity (Sp) were calculated to measure the performance on algorithm comparison:

$$Sn=\frac{TP}{TP+FN},$$

$$Sp=\frac{TN}{TN+FP},$$

$$Acc=\frac{TP+TN}{TP+FP+TN+FN}.$$

## Results

### Feature selection for D prediction

To select the optimal features, the F-score was calculated for each encoding method. Based on the order of F-score, the top N features were used in the five-fold cross-validation. When the top N features achieved the highest performances, more features included in the training will not improve the performances. The results are summarized in Table 2. For *E. coli* D site prediction, 43 features with the highest F-score from the chemical property encoding method show the best performance (AUROC: 0.942). For *S. pombe* prediction, 12 features with decreasing F-score from the nucleic acid composition method achieved the highest AUROC value.

**TABLE 2:** Top *N* features with the highest AUROC.

	*E*. *coli*		*S*. *pombe*		*S*. *cerevisiae*	
	Performance	Top*N*	Performance	Top*N*	Performance	Top*N*
EIIP	0.665	2	0.685	8	0.579	14
autoCor	0.463	9	0.526	5	0.525	4
crossCor	0.595	9	0.554	9	0.543	2
PseKNC	0.645	15	0.656	10	0.550	11
ChemProper	0.942	43	0.771	55	0.847	53
ONE_HOT	0.938	47	0.773	34	0.870	36
CONPOSI	0.752	5	0.774	12	0.653	63
Frequency	0.562	8	0.583	3	0.655	13

**FIGURE 2:**
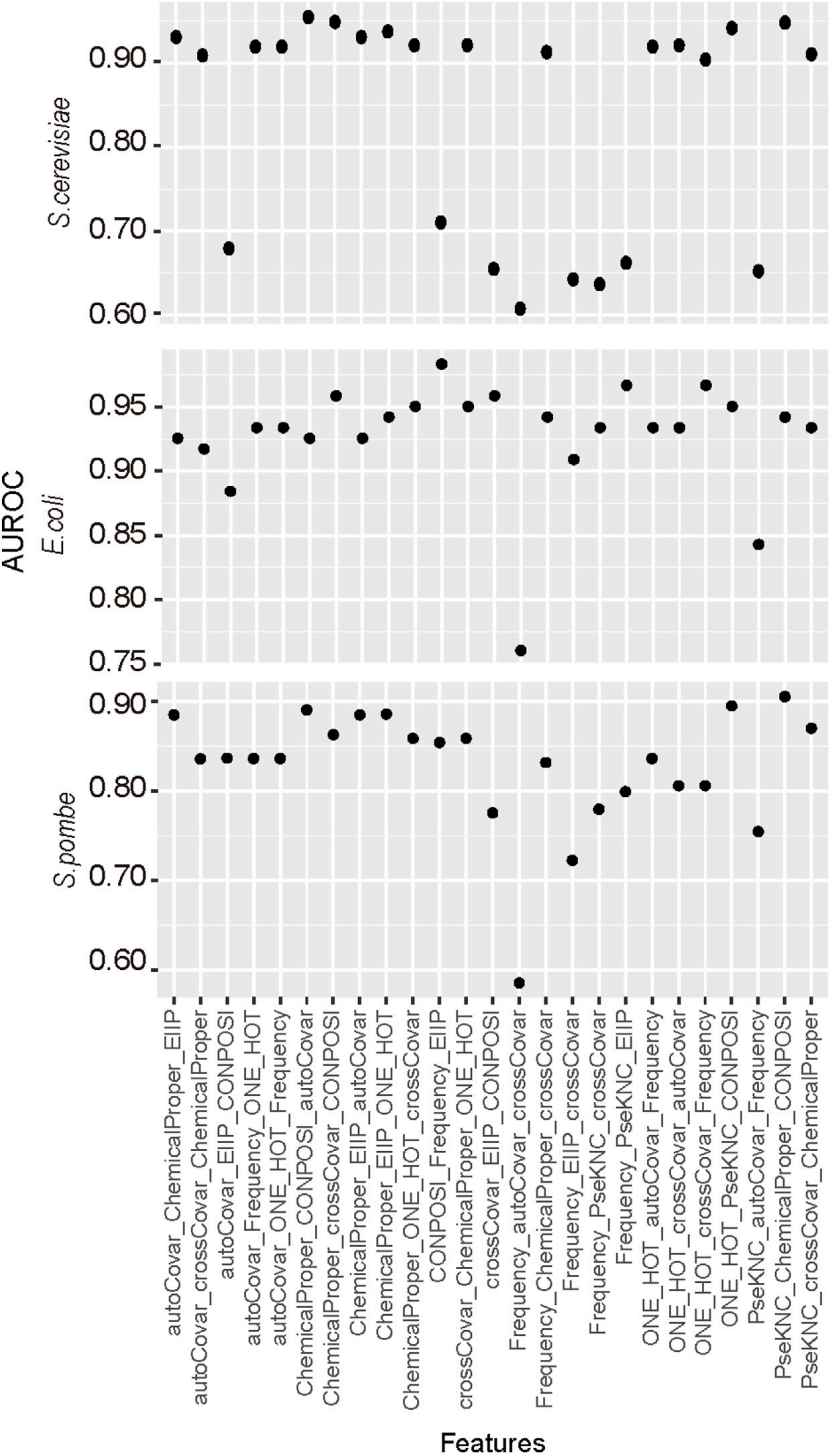
Identification of the optimal combination of feature encoding methods. For each feature, only top *N* features were used in this section, and three different types of features were integrated together.

The feature combination is a common way to improve the prediction performance. In this study, we considered the combination of three types of features. The reason we only considered three rather than more feature types is the limited number of sample sequences as the redundant features may adversely affect the predictor. Different encoding methods with their identified top N features were combined and analyzed by five-fold cross-validation. The results (Figure 2) suggested the best performances for *E. coli* D site prediction were observed when CONPOSI, Frequency, and EIIP were used together, whereas the best choice for *S. pombe* is PseKNC Chemical Proper and CONPOSI. For the D site prediction on *S*. *cerevisiae*, the optimal feature is the combination of Chemical property, CONPOSI, and autoCovar. Interestingly, although using chemical property shows the best performance for *E*. *coli* when one encoding method was used, a combination with more features could not improve its performance.

### Performance comparison among different approaches

**FIGURE 3:**
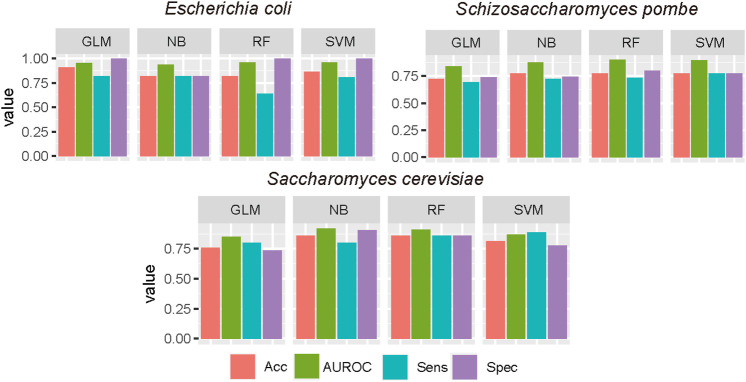
Performance evaluation of different machine learning algorithms. The optimal features were used in different ML algorithms, and the performance was evaluated by the independent test. GLM, generalized linear model; RF, random forest; NB, Naive Bayes.

To evaluate the impact of machine learning algorithms on the D site prediction, besides SVM, GLM, RF, and NB were used to construct predictors. AUROC, ACC, Sn, and Sp were calculated to measure the performance of each algorithm. The results are summarized in Figure 3. Based on the independent test, the performances were stable when different algorithms were used based on optimized sequence features. SVM shows the best performances in *E. coli* and *S. pombe*, while the RF model achieved best performances in *S. cerevisiae*.

### Parameter analysis

**FIGURE 4:**
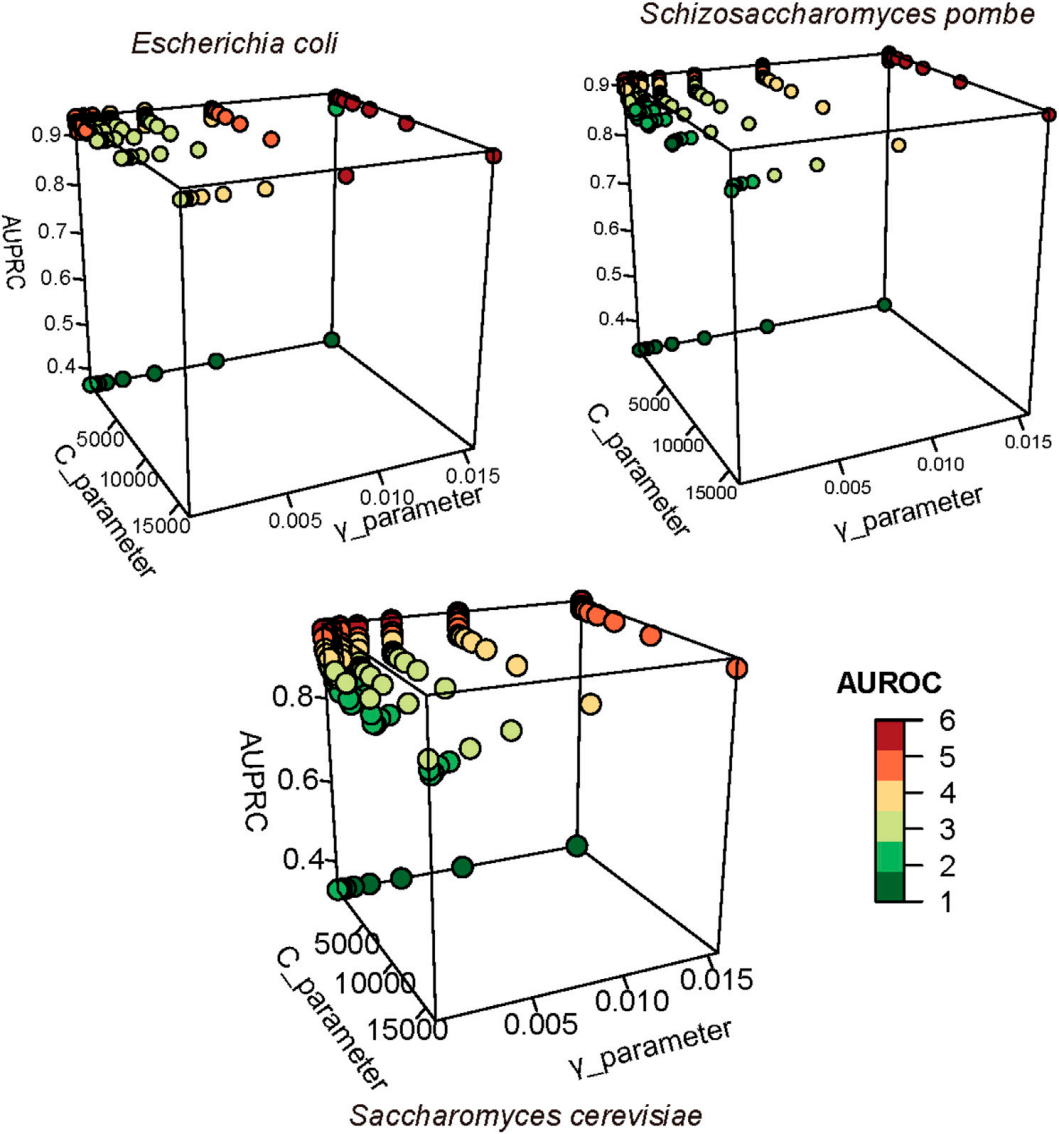
Optimized parameters in SVM. AUROC and AUPRC were used to evaluate the performance of SVM with different parameters. We used different colors to present the number of AUROC; the high value is represented in red, and the low value is represented in green.

The regularization parameter C and the kernel width parameter γ in SVM were analyzed in this study to find the optimal model (Figure 4). For the *S. pombe* D site prediction, when parameter C equaled to 2 ^ (−1) and γ equaled to 2 ^ (−6), the model achieved the best performance with AUROC and AUPRC scores of 0.905 and 0.917, respectively. For *E. coli* prediction, the optimal model can achieve an AUROC score of 0.946 and an AUPRC score of 0.938, when C and γ settings were 2 ^ (−2) and 2 ^ (−9), respectively. For the *S. cerevisiae* D site prediction*,* when C is 2 ^ (−2) and γ is 2 ^ (−7), the predictor achieved the best performance with AUROC 0.955 and AUPRC 0.962.

### Cross-species prediction and data interpretation

**FIGURE 5:**
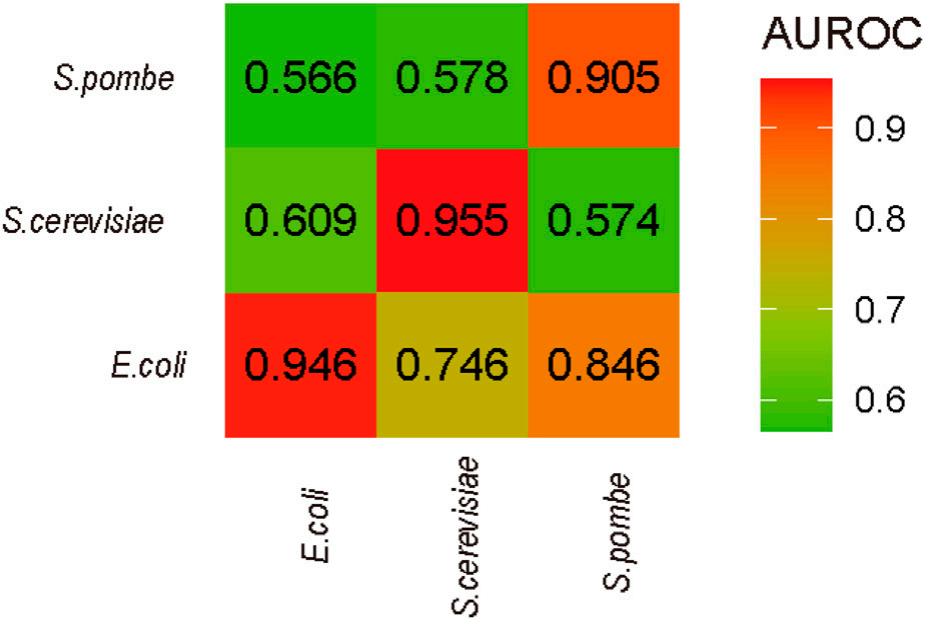
Cross-species prediction. The names of *x*-axis are the species of training data in prediction, and the names of *y*-axis are the species for testing.

To estimate the consistence of D among the different species, the performances of cross-species prediction were used (see Figure 5). The prediction between *S. pombe* and *E. coli* is higher than that between *S. pombe* and *S. cerevisiae*, which is from the same genus. Considering the D sites identified on *S. pombe* and *E. coli* by Rho-seq, the lower performance may be limited by technique preferences, which is a common issue in the RNA modification sequencing field. Additionally, the optimal features of each species were identified, and these specific features may only help for prediction in same species rather than cross-species prediction.

**FIGURE 6:**
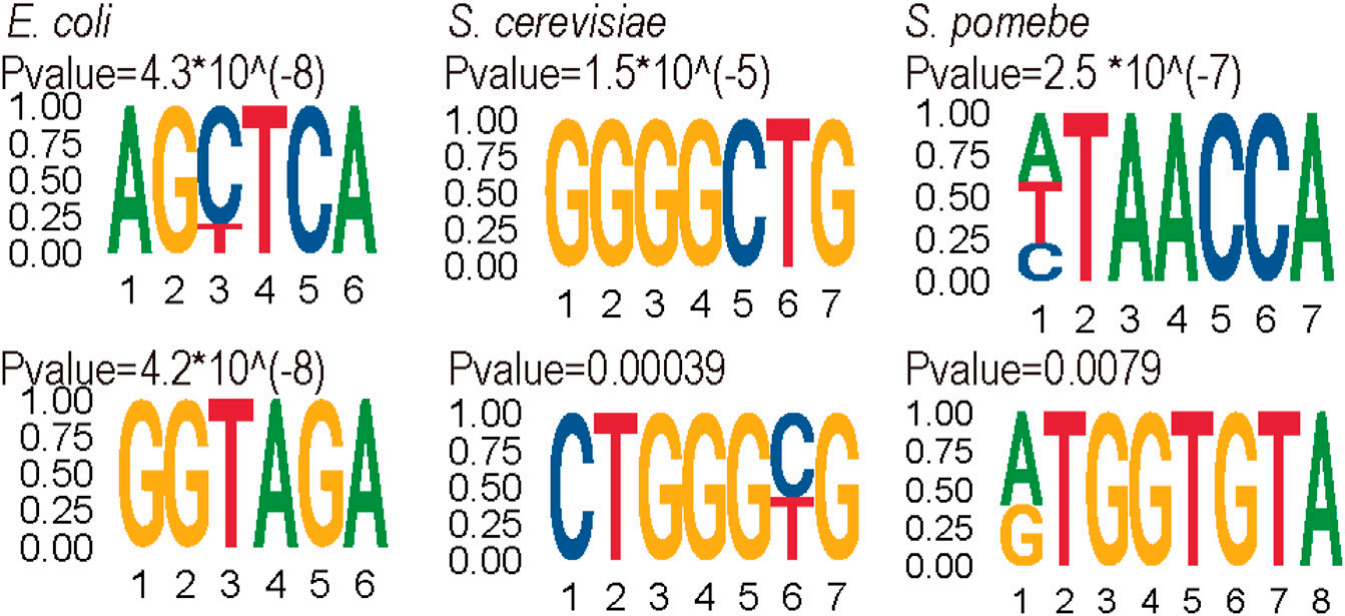
Top two motifs of positive sites by MEME.

Furthermore, the motifs of positive data were analyzed by the MEME suits (Bailey et al., 2015) website (see Figure 6). The results showed the motif of each species is quite different. The motif of *S. cerevisiae* is enriched in the high G contact region, whereas *S. pombe* and *E. coli* are enriched in the ‘GA’ region.

## Discussion

The importance of RNA modifications has been illustrated in the past 10 years, which participates in many biological processes, including stem cell/embryo development, immunity of infection, and carcinogenesis. Additionally, multiple RNA modifications have been proven to be conserved in the evolution. D, as the second abundant tRNA modification, has many molecular functions due to its unique structure and participates in different biological processes. Recent studies have suggested the D modification also appears in mRNA.

With accumulated sequencing results, bioinformatics research studies become an important part of epitranscriptome analysis, which included the peak calling method (Meng et al., 2013; Meng et al., 2014), databases (Liu et al., 2018; Tang et al., 2021; Ma et al., 2022), annotation (Zheng et al., 2018; Chen et al., 2021a), and prediction tools (Chen et al., 2016; Yang et al., 2018; Chen et al., 2019b; Chen et al., 2019c; Feng and Chen, 2022; Jiang et al., 2022; Zhang et al., 2022); all of these provide a convenient way to understand epitranscriptome regulation. In this study, we provided a bioinformatics framework named “DPred_3S″ to predict D sites in *S*. *cerevisiae, S. pombe*, and *E. coli*.

Compared with previous studies (Table 3), we used a new dataset using high-throughput sequencing techniques Rho-seq and D-seq, which provide more D sites in more RNA types rather than tRNA only. After system evaluation, the optimal features and parameter were identified in our work. The high performances of our model suggest the D sites can be distinguished based on their surrounding sequence.

**TABLE 3:** Comparison with other tools.

Tool	Species	RNA type	Technique	Reference
DPred_3S	3	Epitranscriptome	Rho-seq and D-seq	
iRNAD	5	tRNA	Mass spectrum	Xu et al. (2019)
DPred	1	tRNA	Rho-seq	Wang et al. (2023b)

The current study only considered the sequence-derived features, and more advanced encoding methods (Chen et al., 2019a; Huang et al., 2022) could be used to improve the performance in further study. Moreover, deep learning-based algorithms should be integrated to illustrate sequence characteristics by data interpretation.

## Data Availability

The original contributions presented in the study are included in the article/Supplementary Material; further inquiries can be directed to the corresponding author.
